# Specific 50-kHz vocalizations are tightly linked to particular types of behavior in juvenile rats anticipating play

**DOI:** 10.1371/journal.pone.0175841

**Published:** 2017-05-03

**Authors:** Candace J. Burke, Theresa M. Kisko, Hilarie Swiftwolfe, Sergio M. Pellis, David R. Euston

**Affiliations:** 1 Dept of Neuroscience, Univ. of Lethbridge, Lethbridge, AB, Canada; 2 Behavioural Neuroscience, Experimental and Biological Psychology, Philipps-University of Marburg, Gutenbergstr. 18, Marburg, Germany; Texas Christian University, UNITED STATES

## Abstract

Rat ultrasonic vocalizations have been suggested to be either a byproduct of physical movement or, in the case of 50-kHz calls, a means to communicate positive affect. Yet there are up to 14 distinct types of 50-kHz calls, raising issues for both explanations. To discriminate between these theories and address the purpose for the numerous 50-kHz call types, we studied single juvenile rats that were waiting to play with a partner, a situation associated with a high number of 50-kHz calls. We used a Monte-Carlo shuffling procedure to identify vocalization-behavior correlations that were statistically different from chance. We found that certain call types (“split”, “composite” and “multi-step”) were strongly associated with running and jumping while other call types (those involving “trills”) were more common during slower movements. Further, non-locomotor states such as resting and rearing were strongly predictive of a lack of vocalizations. We also found that the various sub-types of USVs can be clustered into 3–4 categories based on similarities in the way they are used. We did not find a one-to-one relationship between any movements and specific vocalizations, casting doubt on the motion byproduct theory. On the other hand, the use of specific calls during specific behaviors is problematic for the affect communication hypothesis. Based on our results, we suggest that ultrasonic calls may serve to coordinate moment-to-moment social interactions.

## Introduction

Rats and related rodents emit a large number of ultrasonic vocalizations in the 50-kHz range [1]. There have been two major hypotheses advanced to account for these calls: the movement byproduct hypothesis and the affective communication hypothesis. The movement byproduct hypothesis posits that the 50-kHz vocalizations emitted by rodents are largely produced by the biomechanical forces on the thoracic area generated during movement [2]. In contrast, the affective communication hypothesis posits that these vocalizations convey the animal’s affective state to surrounding animals. The fact that 50-kHz calls are highly varied, with some authors recognizing as many as 14 different kinds involving distinct patterns of frequency modulation (FM) [3] raises questions about how the two hypotheses can account for such variability.

Thiessen and collaborators pioneered the movement byproduct hypothesis after finding a strong correlation between locomotion and the production of some ultrasonic vocalizations (USV) in gerbils [4]. Particularly striking was that the calls were emitted most often when the animal landed on its forefeet when jumping, when the lungs are presumably compressed and air expelled. Similarly, vocalizations have been linked to the landing phase of hops and darts in female rats during mating [2]. Vocalizations necessarily occur on the expiratory phase of respiration and hence, behaviors which modulate breathing could also modulate USVs [5]. For example, USVs are frequently seen during sniffing behavior and show periodicity in the 6–8 Hz range, synchronous with the sniffing respiratory rhythm [6]. Locomotor movements are known to entrain respiration in many quadrupeds, including rats [7, 8]. Hence, it is plausible that vocalizations are entrained to locomotion.

Several lines of evidence argue against the movement byproduct hypothesis. First, although sniffing and walking entrain respiration, rats can sniff and move without vocalizing [6, 8]. In fact, electromyographic studies suggest that vocalizations require the active contraction of the larynx muscles during the exhalation [9, 10]. Concordantly, rats with transected laryngeal nerves do not vocalize even though they actively move about [11, 12]. Second, respiration and locomotion can be decoupled during vocalizations as the rat prolongs the expiratory phase to produce a call [8]. Third, the periodic frequency modulations produced during trills require active oscillatory adduction of the laryngeal muscles, suggesting that complex calls are not produced by accident [5, 9]. Fourth, a recent study reported that vocalizations often occur at the onset of locomotion, not when the animal is stopping [13]. Finally, the presence of another rat can influence vocalization rate independent of the rate of movement [13]. Despite this evidence, it is clear that vocalizations are strongly associated with movements, leaving open the possibility that at least some vocalizations are motion byproducts [8, 13].

The alternate hypothesis is that USVs are indicators of affective state [14], with 22 kHz calls indicating negative affect and 50-kHz calls indicating positive affect. The 22kHz calls are emitted in situations where the rat may experience anxiety [1]. For example, 22 kHz calls are elicited by predators (such as a cat) [15], foot shock [16], fear conditioning [17–19], a dominant conspecific [20–22], acoustic startle [23], handling by an unfamiliar handler [24, 25], and social defeat [21, 22]. In contrast, 50-kHz calls are present during anticipation of rewards such as an addictive drug (amphetamine or methamphetamine) [26], sexual encounters [27], stimulation of brain reward pathways [28], social play [29] and “tickling” by human handlers [30]. Further distinctions have been drawn between different categories of 50-kHz calls, such as flat and frequency modulated (FM) calls [25–28]. Flat 50-kHz calls have been proposed as a social coordination signal based on the finding that rats in their home cage emit these calls when a partner is removed [31]. These calls are also associated with aggressive interactions and, therefore, could be an indicator of social ambivalence [29]. In contrast, FM calls have been associated with a variety of positive situations such as social interactions or rewards [26–28]. However, the affective communication hypothesis does not predict nor offer a particularly compelling account for why there should be as many as 14 categories of vocalizations.

One of the issues complicating the understanding of ultrasonic calls is that it is not yet clear where to draw categorical boundaries between calls. As mentioned, a distinction is often made between flat and FM calls [3, 29, 31, 32]. FM calls are sometimes further subdivided. For example, Takahashi et al. [33] divided FM calls by the frequencies around which they range (25kHz, 40kHz or 60kHz). The most extensive categorization scheme is that proposed by Wright et al. [3] who identified 14 distinct categories of 50-kHz calls based on acoustic parameters and call profile. Similarly detailed schemes have been devised for mouse vocalizations [34, 35]. However, it has been argued that these categorization schemes are more detailed than necessary [36]. Exactly which call categories are needed to capture the essential features of rat communication remains an open question.

In this experiment, we sought to discriminate between the movement byproduct and affective communication hypotheses by examining the correspondence between specific subtypes of 50-kHz calls and specific movements. We examined behavior and vocalizations while juvenile rats awaited the arrival of a play partner, a period associated with a large number of 50-kHz calls [37] which, owing to the lack of a partner, can be uniquely ascribed to one animal. The movement byproduct hypothesis predicts that vocalizations should be tightly correlated with actions involved in locomotion, not those in which the rat is stationary. Further, because each movement produces its own characteristic airflow pattern, there should be a one-to-one correspondence between specific movements and vocalization types. The affective communication hypothesis, on the other hand, simply predicts a high number of positively valenced calls, presumably intended to elicit play from the expected partner. In this view, calls need not be associated with any particular behavior. Hence, a finding that 50-kHz vocalizations are decoupled from behaviors would strongly favor the affective communication hypothesis over the movement byproduct hypothesis.

## Methods

### Ethics

All procedures reported here were in accordance with the Canadian Council on Animal Care guidelines. This study was specifically approved by the University of Lethbridge institutional Animal Welfare Committee.

### Subjects

A total of 36 male, juvenile Long Evans rats were used in this study. All animals were bred at the Canadian Centre for Behavioral Neuroscience at the University of Lethbridge and were housed in pairs until testing. Nine subjects were used in Analysis 1 and 12 subjects in Analysis 2. The remaining 18 animals were cage-mates of the target animals, whose data only became relevant during social interactions, not reported here. Testing occurred from post-natal days 26–38, spanning the age when play is most frequent (30–40 days from birth) [38].

### Behavioral procedure

#### Apparatus

The testing enclosure was a Plexiglas box (50x50x50 cm), which was situated inside a soundproof chamber (61x61x83 cm) lined with acoustic foam. The floor of the chamber was covered with 2 cm of paper-based bedding (Softzorb, Nepco, Warrensburg, NY) which we found to facilitate play while causing very low levels of ultrasonic interference. Ultrasonic vocalizations were collected using a specialized microphone (Model 4939, Brüel & Kjaer, Denmark) with a frequency response of 4-Hz to 100-kHz. The microphone was located in the ceiling of the chamber and was approximately 15 cm above the middle of the play enclosure. The microphone was connected to a Soundconnect™ amplifier (Listen, Inc, Boston, MA) and sound waves were recorded at 195,313 Hz using 16-bit resolution via a multifunction processor (model RX6, Tucker-Davis Technologies, Alachua, FL). Video was recorded using two cameras. One was a USB webcam (Microsoft Lifecam Studio, Redmond, WA) with its infrared filter removed, positioned directly above the animal and the second was a Sony digital video camera, also with infrared sensitivity, with a view through the side of the testing chamber at a downward angle of 45 degrees. Illumination was provided by an array of 16 100 mW infrared LEDs (Osram SFH 4557, Regensburg, Germany) mounted on the ceiling of the soundproof chamber and from the in-built infrared light source in the Sony camera.

#### Anticipation of play test

Data presented here were taken from a 2-minute anticipation period during which a target animal waited in the testing enclosure for the arrival of a familiar play partner (i.e., his former cage mate). Once the play partner was introduced, then animals were allowed to play for 5 minutes, following previously established methods [39]. Prior to testing, pairs were habituated to the enclosure for 30 min each day for 6 consecutive days. On the 6^th^ day all subjects were socially isolated from their cage mates for 24 hours prior to play testing and isolation continued until after all 7 days of testing were complete, in order to increase overall playfulness [40–42]. Both habituation and testing sessions occurred immediately at the end of the light cycle and were conducted in complete darkness, as this has been shown to facilitate USV production [37]. Audio and video recordings began after the target rat was placed in the test enclosure. Because both audio and video data were recorded on separate devices, a custom-made beeper with an LED light was used to emit a simultaneous light/sound cue at the beginning and end of each recording session and these times were used to synchronize audio and video recordings during subsequent analysis. Following each session, rats were returned to their home cages, and the apparatus was thoroughly cleaned with Virkon, a broad-spectrum disinfectant (Virkon Disinfectant Technologies, Sudbury, United Kingdom), and bedding was replaced to avoid any odors from other subjects.

### Scoring

#### Behavior

For each animal the type of behavior and duration of that particular behavior were scored during the 2-minute testing session. Importantly, we assigned a behavioral category at every time point, so that no time was left unaccounted for. Knowing the rats’ behavior at every time point was important because in our analysis, that lack of a vocalization during a specific behavior was informative (e.g., a call which occurs for 8 of 8 instances of a behavior is more tightly coupled to that behavior than one which occurs only 3 times out of 8). Behaviors were coded via frame-by-frame analysis of video taken either at a 45-degree angle relative to vertical or via an overhead camera. A switch from a camcorder (45 degree angle) to an infrared webcam (overhead) occurred mid-way through data collection due to the greater ease-of-use of the webcam. We have found that either view is sufficient to distinguish the behaviors we studied here.

The behaviors included in the analyses are described in Table 1, which also describes the criteria used in categorization. Separate coders performed behavioral (and vocal) coding for each of the analyses. In the first analysis, overlapping behaviors were allowed (e.g., if the animal turned while walking, that time interval would be coded as both a turn and a walk). In the second analysis, each time interval was assigned only to one behavior, thus simplifying our vocal-behavioral correlation analysis. In the case of turning and walking simultaneously, the behavior was coded as a walk. Most other behaviors were mutually exclusive by their nature.

**Table 1:** Description of Rat Behaviors.

Behavior	Description
Step	Removal of at least two paws from the ground in an alternating manner
Walk	Removal of all four limbs off the ground in an alternating manner (left paw and right hind limb move simultaneously followed by right paw and left hind limb) OR significant shift from one location to another (if all limbs are not visible)
Run	Only two limbs touch the ground at any given time; the rat may alternate two limbs at a time (as is seen during walking behaviour) OR the rat may move two paws followed by two hind limbs at any given time; such movement is accompanied by the extension of the torso as the front limbs reach forward followed by flexion of the torso as the hind limbs are removed from the ground and placed under the body [43]
Jump	Up jump: the two front limbs leave the ground followed by the hind limbs while body is lifted into the air, then all limbs touch the ground simultaneously or closely one after the other [44]; Forward jump: the two front limbs are extended forward and removed from the ground followed by the removal of the hind limbs from the ground; this behaviour is accompanied by the extension of the torso as the front limbs reach forward followed by flexion of the torso as the hind limbs are removed from the ground
Turn	Turn with one or both front limbs at a 45-, 90-, or 180-degree angle OR turn with three or more limbs at a 360-degree angle. Turning may also be preceded by a stepping or walking pattern or followed by a rear (see below for the operational definition of rearing behaviour)
Explore	Head may move up and down or side to side, usually includes whisking; may include extension of paw but no net ambulatory movement.
Dig	Vigorous forward and backward motion of front limbs while significantly displacing bedding
Rear	Standing on rear limbs with both front paws off ground (either free standing or against wall)
Shake	Vigorous side-to-side shudder of head, neck, and trunk [45]
Groom	Licking of paws; wipes/rubs face and nose; wipes behind ears, neck, and/or downward to either side of the body; may grab fur and nibble with teeth. Grooming may consist of a variation of these behaviours many consecutive times. However, grooming is typically initiated by wiping of the nose or face and followed by grooming of the neck and body [46]
Scratch	Rapid movement of hind limb with the claws rubbing against head, neck, or side
Rest	Immobile; may include slow head movements but feet do not move.

#### Vocalizations

**Fig 1:**
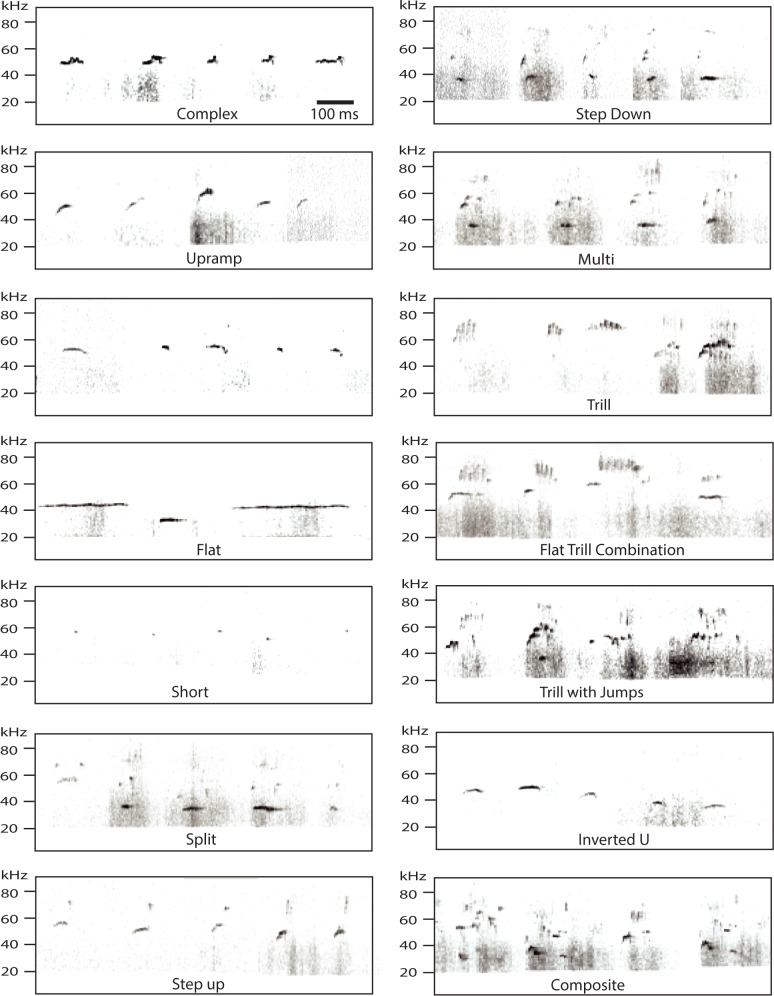
Examples of categorized 50-kHz calls (as originally described by Wright and colleagues [3]) taken from our data.

Acoustic data was analyzed using Raven Pro 1.4 software (Bioacoustics Research Program, Cornell Lab of Ornithology, Ithaca, NY). The Raven Pro software generated spectrograms with a 256-sample Hann window from which the experimenter manually selected 50-kHz vocalizations. 50-kHz vocalizations were included if they followed Wright et al.’s [3] call criteria, specifically: 1) temporal continuity, 2) fundamental frequencies between 35- and 90-kHz, and 3) a local peak in sound intensity distinct from background noise that exhibited patterned temporal structure. Some putative vocalizations were not perceptually distinct from the background noise and were hence removed from further analyses. After manual scoring, the Raven Pro software provided the beginning and end times of each vocalization as well as the manually determined category of each call. The call classifications were done according to the 14 categories identified by Wright et al. [3]. Although these calls were identified from slightly older rats (60–70 days of age), most of our calls were highly similar to at least one of the 14 adult call types, suggesting that the call structure of Long-Evans rats does not change much between 1 and 2 months of age. Examples of vocalizations in each category are shown in Fig 1. As with behavioral coding, categorization of vocalizations for each experiment was by one experimenter and the coders differed for analysis 1 and 2.

### Analyses

#### Overview

We present data from two separate analyses (meaning a full coding of all behaviors and vocalizations) taken during anticipation of play in 18 rats. The purpose of the first analysis (Analysis 1) was to explore the feasibility of the analysis methods. It included data recorded at multiple time-points in the experiment (testing days 1, 5, 6, and 7) and, in some cases, included two data sets from the same animal because that is the data that we had available at the time. In light of the results of the first analysis, a second, better controlled, analysis (Analysis 2) was conducted by a different researcher (C.B.) using only data from day 7 of testing and with each animal contributing only one session. Day 7 was used because this is when the most vocalizations occurred and it is also the session most likely to have calls of a social nature (relevant to the affective communication hypothesis). Three animals from the first analysis were included in the second, but using different sessions so that there was no overlap between the data used in the two analyses. As part of Analysis 2, we also quantified the total number of calls and time spent in locomotor behaviors (turning, walking, running and jumping) between the first and last day of training (Day 1 vs Day 7).

#### Monte-Carlo shuffling

The total number of behavioral and vocal events differed substantially between different categories, meaning that a simple correlation of behaviors with vocalizations would have been problematic to interpret. For example, there were a very large number of trills that occurred during walking, but this could be simply because trills were the most common vocalizations and walking was one of the most common behaviors. A statistical analysis that accounted for these differences in event baselines was necessary to determine whether a particular vocalization co-occurred with a particular behavior above chance levels. Therefore, a Monte-Carlo technique was used to calculate the probability distribution of each unique combination of vocalizations and behaviors. We first counted the number of co-occurrences of each vocalization type with each of the coded behavioral categories. Next, for each session, vocalizations were repeatedly shuffled and the number of behavior-vocalization co-occurrences computed. A vocalization was counted as occurring during a particular behavior if the mid-point of the call occurred between the start and stop time of the behavior. To allow for errors in the coding of the behavior times, we expanded the time window for each behavior by 200 ms in each direction. Shuffling was achieved by assigning each vocalization a random time within the duration of the 2-minute observation period. Hence, the relative frequency of vocalizations was kept the same for each shuffle. This shuffling was done 10,000 times and the total number of co-occurrences of each vocalization type with each type of behavior was tabulated. Based on the distribution of these counts, we assigned a z-score to the actual number of occurrences. The higher the z-score, the less likely that specific combination of vocalization and behavior could have occurred by chance (i.e. for the number of sessions included here, a z-value of 2.8 gives a *p*-value of <0.05 and a z-value of 3.3 gives a *p*-value of <0.01). Shuffling was performed separately for each session and the z-scores averaged across session to generate the final, average z-score values.

#### Proportion of movements associated with vocalizations

To further assess the link between movement and vocalizations, we measured the proportion of times that each movement type was accompanied by a vocalization. A 200 ms window was created, centered on the onset or offset of each movement (i.e., 100 ms before to 100 ms after the movement onset or offset). We then tabulated the number of times each vocalization type overlapped with this window. For this analysis, each vocalization type was counted either as an occurrence or non-occurrence (i.e., two trills before a walk would be treated as a single occurrence). The results were expressed as a proportion of the total number of movements. For example, if there were 80 instances of the movement type “run” and 60 of them were accompanied by a “trill” call during the onset of that movement, we reported that 75% of runs began with a trill. The total proportion of movements accompanied by a call was calculated by summing the proportions of co-occurrence across each of the vocalization types.

#### Dendrogram of vocalization similarity

To assess the similarity of vocalizations based on their behavioral correlates, we created a dendrogram. Similarity was based on the z-score matrix describing the relative frequency of occurrence of each vocalization and behavior, as described above under Monte Carlo Shuffling, with vocalizations in rows and behaviors along the columns. We measured the distance between each pair of rows of this matrix as 1-*r* where *r* is the correlation between rows. A distance of near zero meant that the behavioral correlates for those two vocalizations were nearly identical whereas a distance of 1 meant that the behavioral correlates were unrelated. Values above 1 indicated that the behavioral correlates were anti-correlated. A dendrogram was then generated from this data using the Unweighted Pair Group Method with Arithmetic Mean (UPGMA) algorithm, implemented in the BMEToolbox (http://www.bioinformatics.org/mbetoolbox/) [47] written for Matlab (The Mathworks, Natick, MA).

#### Student’s t-tests

A 2-tailed paired *Student’s t-test* was used to assess whether there was a significant increase in vocalizations form day 1 to day 7 for all animals in Analysis 2. Each rat contributed two measures to this analysis, one for the total number of calls during the two-minute anticipation period on day 1 and another for the total number of calls in the same period on day 7 of training. A similar analysis was performed on the total amount of time spent in locomotor behaviors. Time was used rather than counts because behaviors are extended over seconds in contrast to vocalizations which are so short that they can be considered discrete events

#### Inter-rater reliability analysis

To assess inter-rater reliability of both behavioral and vocal data, data from three sessions were coded by both of our coders. To compute reliability, each session was broken down into 10 ms bins and the behavior or vocalization within that bin tabulated for each coder using an arbitrary numbering scheme (e.g., turn was assigned a 1, walk was assigned as a 2, and so on). We then ran a simple correlation between the ratings of the two coders, including all bins from all three sessions. In the case of vocalizations, bins with no vocalization coded were excluded before running the correlation because there were many long stretches of silence. Note that judicious assignment of numbers to behaviors or vocalizations (i.e., giving similar calls/behaviors closer numbers) would improve the reliability ratings, so the reported values should be considered a conservative estimate of inter-rater reliability. Further, independent of the numbering scheme, a correlation of one would always mean total agreement.

## Results

### Vocalization counts during anticipation of play

To investigate the behavioral correlates of vocalizations, we ran juvenile rats in an anticipation of play paradigm, analyzing vocalizations during the two-minute period before the introduction of a play partner. Animals were tested over the course of 7 anticipation periods, one per day, each followed by pairing with the same play partner (see Methods for details). In the first analysis, we used data from 9 animals and a total of 14 play anticipation sessions (some animals contributed two sessions recorded on different days). To test the robustness of our results, a second analysis was conducted, using data from 12 rats and a total of 12 test sessions, all from testing day 7.

**Fig 2:**
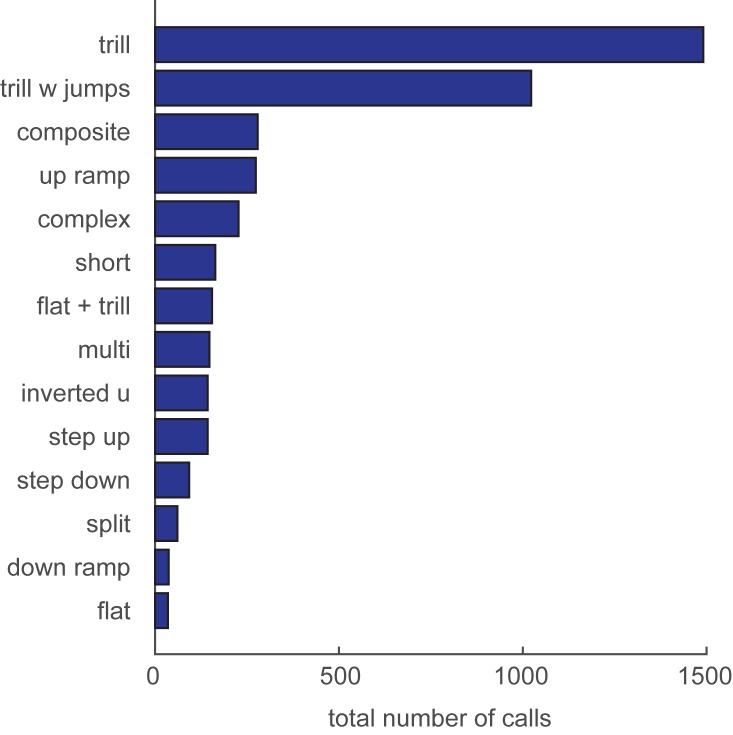
Total number of vocalizations by call category. Categories correspond to those shown in Fig 1. Bars indicate the total raw counts of vocalizations across Analysis 1 and Analysis 2 combined.

Previous studies have shown that FM 50-kHz calls are most common during appetitive situations. For the purposes of comparison with previous studies, we counted the frequency of occurrence of each of the 14 call sub-types identified in this study, using categories previously suggested by Wright et al. [3]. As shown in Fig 2, calls containing trills (trills, trills with jumps, and composites) were by far the most frequent types of calls. In comparison, flats were the least frequent call. These data contrast with those of Wright et al, who found that almost half of the calls emitted by young adults tested one at a time were flats, but the data agrees with Himmler et al. [48], who found that the most frequent vocalizations during play by juveniles were trill type calls, accounting for 77% of the total calls.

### Training effects on vocalizations and behaviors

To address predictions made by the locomotor byproduct and affective state hypothesizes, we examined changes in vocalization and behavior prevalence between training days 1 and 7. First, we compared the overall number of 50-kHz calls on both day 1 and day 7 for all rats included in analysis 2 (analysis 1 could not be used due to missing data). A 2-tailed paired *Student’s t-test* showed a significant increase in 50-kHz calls from day 1 (M = 92.9 calls; SD = 82.6) to day 7(M = 182; SD = 95.3; t = 3.04, df = 14, p = 0.009), suggesting that at least some of the calls observed were due to anticipation of a play partner. Could increased movement account for the increased number of vocalizations? To address this, we compared the total time spent in locomotion (walking, running or jumping) between day 1 and day 7. A repeated measures *Student’s t-test* revealed that locomotion time actually decreased with training (Day 1: M = 56.7 sec; SD = 19.1; Day 7 M = 28.4 sec; SD = 10.6; t (10) = 5.59, p < .001 (2 tailed)). On the other hand, the total number of jumps and runs both increased significantly from day 1 to day 7 (Jumps: Day 1: M = 2.00; SD = 2.57; Day 7: M = 9.18; SD = 6.27; t (10) = -3.70, p = .004 (2 tailed); Runs: Day 1: M = 0.64; SD = 1.03; Day 7: M = 7.27; SD = 7.48; t (10) = -2.85, p = .017 (2 tailed)). In other words, rats trained to expect a play partner spend less overall time moving, but those movements they do make are more vigorous. Because both vocalizations and locomotion increase with training, these data do not discriminate between the locomotor byproduct and affective state hypotheses.

### Specific correspondences between vocalizations and behaviors

To further address the locomotor byproduct and affective state hypothesizes, we counted the number of co-occurrences of each vocalization type with each of the coded behavioral categories. A Monte-Carlo shuffling technique was then used to estimate how likely the observed frequency of coincidence between a behavior and a vocalization was to have occurred by chance, expressed as a z-score (a z-score above 2.3 is significant at p < .01). Negative z-scores indicated that the call was much less likely to occur during the specified behavior than if calls were randomly distributed.

**Fig 3:**
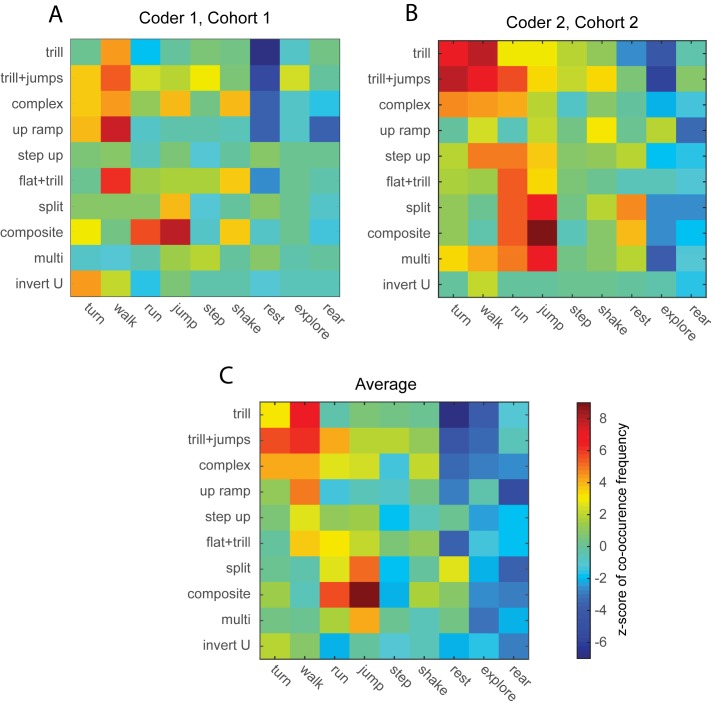
Z-scores showing the probability of occurrence of each of the vocalization-behavioral combinations are graphically presented. (A) Data from Analysis 1. (B) Data from Analysis 2. (C) Grand average of the results from Analysis 1 and 2. Colors indicate the z-score and the same color scheme is used for all subplots. Note that vocalizations and behaviors with very low rates of occurrence have been omitted, as it was not possible to run statistical analysis for these low-frequency events.

As shown in Fig 3, some actions are clearly more likely to be associated with certain vocalizations than others. Our first analysis was a preliminary study to establish the feasibility of the method, and hence the behavioral sessions were taken from different days during testing. Despite this variability, the z-scores from this study show clear evidence of certain calls being tied to certain behaviors. As shown in Fig 3A, jumping behavior is consistently associated with the composite, split, and complex call categories with *z*-values of 7.9, 3.7, and 3.7 respectively. Running appears to be consistently associated with the composite and trill-with-jumps calls. Walking was associated with the trill-with-jump, trill, complex and flat-with-trill calls. Further, several calls were associated with turning (invert U, trill-with-jumps, complex and ramp) and shaking (complex, flat-with-trill, and composite). Finally, rearing, resting, and exploring are among the least likely behaviors to be coupled with any type of vocalizations. Z-scores for the most statistically significant vocalization-behavior correspondences are shown in Table 2.

**Table 2:** Ten strongest associations between behaviors and vocalizations are shown for Analysis 1. Values are z-scores.

Association Type	*z*-value
Jump–Composite	7.9
Walk—Upward Ramp	7.6
Walk—Flat-Trill Combo	6.1
Walk—Trill with Jumps	5.5
Walk—Step Down	5.5
Explore—Downward Ramp	4.6
Walk—Trill	4.4
Turn–Inverted U	4.4
Shake–Composite	3.8
Turn–Upward Ramp	3.8

A second analysis was conducted to test the reliability of our methodology and to insure that our results actually reflected calls emitted during the anticipation of a play partner. While analysis 1 focused on a variety of testing days, the second analysis used data from 12 rats on their 7^th^ day of testing. As the vocal and behavioral scoring for each analysis involved a different coder, an inter-observer reliability analysis was conducted. To do this, the two experimenters coded three data sets in common. The raw behavioral counts showed a 92% agreement between coders. For vocalizations, the raw vocalization counts showed only a 36% agreement. Upon closer inspection, it was determined that the discrepancies were due to disagreement over how to categorize four highly similar call types: trills, complex, composite and trill-with-jumps. Accounting for this confusion gave 98% agreement in call categorization. Despite the less than complete agreement between coders, the data from analysis 2 (Fig 3B) look strikingly similar to those from analysis 1 (Fig 3A). Again, jumps were highly correlated with composite and split calls, but now also with multistep calls. Runs were now associated with a much wider range of call types, including multi-step, split, flat-trill, step up, and trill-with-jumps. Walking was now more associated with trill and step-up but not flat-trill calls. Turning was now associated with trill but not up ramp calls. Some splits occurred during rest, but again, resting, exploring and rearing were either unrelated to most calls or even anti-correlated. Z-scores for the most statistically significant vocalization-behavior correspondences are shown in Table 3.

**Table 3:** Ten strongest associations between behaviors and vocalizations are shown for Analysis 2. Values are z-scores.

Association Type	z-value
Jump–Composite	8.8
Walk–Trill	8.3
Turn–Trill with Jumps	7.6
Jump–Multi	7.4
Turn–Trill	6.8
Walk–Trill with Jumps	6.6
Jump–Split	6.3
Run–Trill with Jumps	6.0
Run–Split	5.4
Rest–Split	5.4

Given the gross similarities between the results of analysis 1 and 2 we averaged the results (Fig 3C) to present a summary of our findings. Speaking broadly, slower locomotion (turning and walking) is associated with calls that include trills or warbles (trill, trill with jump, and complex) as well as up ramps. As locomotion becomes more vigorous (running and jumping), vocalizations shift to composite and sometimes split and multi-step calls. Again, less active states such as resting, exploration and rearing are associated with a marked reduction in the probability of calls, suggesting that these are receptive states for the rat (i.e., they are quiet, perhaps listening, rather than emitting calls).

**Fig 4:**
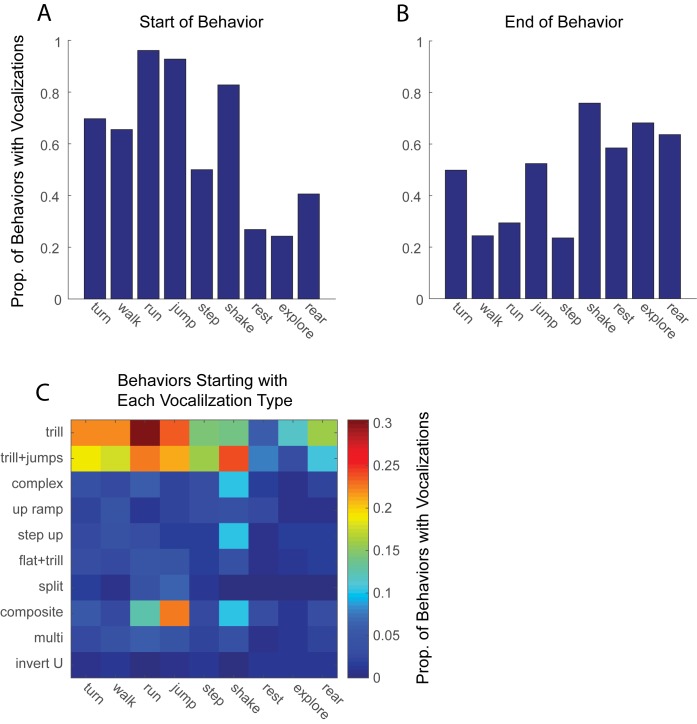
Proportion of each behavior associated with vocalizations. **(A) Bars show the proportion of times each behavior type was accompanied by any vocalization within 100 ms of the onset of that behavior**. (B) Same as A but vocalizations are counted within 100 ms of the offset of the behavior. (C) The proportion of times each behavior was accompanied by vocalization, broken down by the specific type of vocalization.

The highly significant statistical relationship between jumps and composite calls is consistent with the idea that composite calls are a byproduct of jumping. Similarly, calls with trills might be byproducts of walking and running. To investigate these possibilities further, we examined the consistency of the coupling between calls and behaviors, collapsing across data from Analyses 1 and 2. If a call was caused by a particular movement, then we’d expect a one-to-one correspondence between that call (or group of similar calls) and that movement. To address this, we first computed the proportion of times that each behavior was associated with any call. We focused on calls either within 100 ms of the onset or offset of the movement because these are the times of the most dramatic postural change and hence the most likely to lead to thoracic compression and involuntary calls. As shown in Fig 4A, there were three behaviors which were frequently accompanied by calls: runs, accompanied by calls 96% of the time, jumps, accompanied by calls 93% of the time, and shakes, accompanied by calls 83% of the time. In comparison, with the possible exception of shakes, the offset of behaviors was less frequently accompanied by a call (Fig 4B). Note that the relatively high number of calls after a rest, explore and rear are likely onset responses to the subsequent movement, which are often locomotive.

The locomotor byproduct hypothesis also predicts that a given movement should elicit the same (or highly similar) call. As seen in Fig 4C, this is not the case, even for running, which is associated with composite calls 13% of the time, trill with jumps 23% of the time, and trills 30% of the time. As shown in Fig 1, these calls are fairly distinct. Similarly, shakes are accompanied by trills, trills with jumps, complex, step-up and composite calls. These findings are hence not consistent with the idea that the movement obligatorily causes a specific type of call.

### Similarities between calls based on behavioral correlates

**Fig 5:**
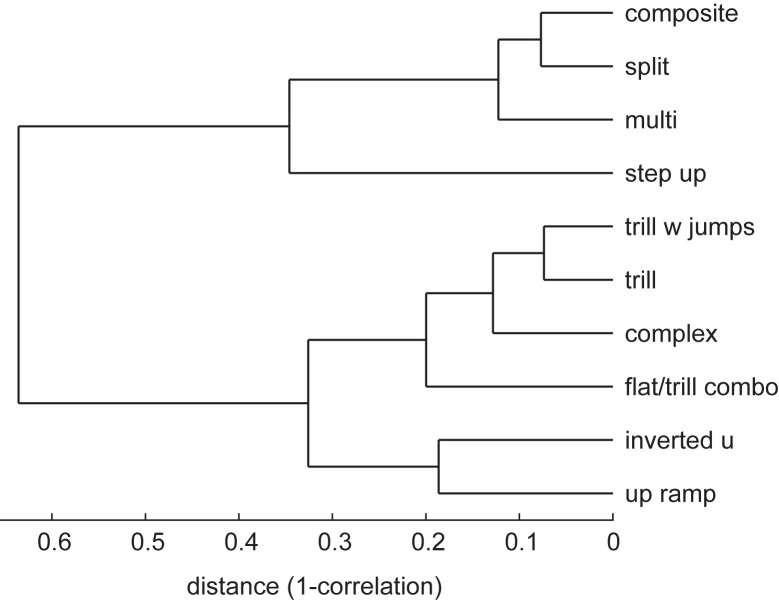
Dendrogram showing the relative similarity of different call types based on behavioral correlates. The x-axis shows the distance between pairs of vocalizations based on the correlation between their behavioral z-scores (i.e., the rows of Fig 3). The dendrogram was generated using the Unweighted Pair Group Method with Arithmetic Mean (UPGMA) algorithm. As with Figs 3 and 4, data from the 4 least frequent vocalizations have been omitted.

It is apparent from Fig 3, that certain calls have similar behavioral correlates. For example, trill and trill with jump calls are almost equally likely to be emitted during turning and walking, whereas split, composite and multi-step calls are all associated with jumping. To assess this further, we performed a clustering analysis, based on the pair-wise correlations of the behavioral z-scores shown in Fig 3C for each vocalization. The results, shown in Fig 5 indicate that the ten most frequent calls types can be grouped into three or perhaps four larger categories. The first consists of composite, split, and multi-step, calls that are generally associated with vigorous activities such as jumping. The second group consists of calls with trills (trill, trill-with-jumps, complex and flat-trill calls), which are generally associated with slower locomotion. A third group consists of the inverted U and up ramp calls, whose behavioral correlates were not well defined. The step-up calls may form a fourth group that seems to be solely associated with walking. As we discuss below, it is important to bear in mind that these results can be influenced by the perceptual similarity of the calls. Two calls that are often confused by coders will end up having highly similar behavioral correlates. However, they also point the way to a more objective method for determining a categorization scheme that reflects the natural communicative purposes used by rats.

## Discussion

Our results show that specific sub-types of rat 50-kHz vocalizations are strongly associated with specific behaviors during play anticipation. Vocalizations frequently co-occurred with active behaviors, such as running, walking and jumping. Less active behaviors, such as exploratory sniffing and rearing were strongly predictive of a lack of any vocalizations. Our findings shed light on two prominent hypothesizes thought to account for the emission of 50-kHz calls: the locomotion byproduct hypothesis [2] and the affect state hypothesis [1, 36, 49].

The motor byproduct hypothesis predicts a much more specific relationship between movements and vocalizations than we have observed. Certainly, locomotor behaviors were far more likely to be accompanied by a call than stationary behaviors. Further, a closer inspection showed that three vigorous movements, runs, jumps and shakes, were almost always (83–96%) associated with a call and these calls occurred at the onset of the movement. However, if the calls are a consequence of the movement, the similar mechanism of production should cause all calls associated with a particular movement to be highly similar. This was not the case. Runs, for example, were strongly associated with trills, trills with jumps, and composite calls. If calls are a physiological by-product the relationship between body movements and vocalization must be very complex. Our data strengthen the arguments made by Laplange et al. [13], discussed in the Introduction, against the notion that vocalizations are physiological coupled to movement.

Consistent with the idea that 50-kHz calls communicate positive mood, rats emitted more of these calls when prior experience led them to expect a play partner. This is consistent with previous reports [13, 37]. Rats also exhibited more runs and jumps under these conditions. The relationship between calls and movement in this case might arise not because the locomotion is causally producing the calls, but because both locomotion and calling are indicative of elevated positive affective. This explanation is consistent with the effects of amphetamine and cocaine, which elicit robust increases in 50-kHz vocalizations and activity levels [50, 51], with high frequencies of calls being emitted whether the rats are moving or not [52]. On the other hand, the affective communication hypothesis offers no explanation for the specific relationships between calls and actions shown in Fig 3. It might be the case that rats shift from trills to composite calls to communicate both increasing speed and positive mood. But ultimately, this is a *post hoc* explanation.

If neither the motion byproduct nor the affective communication hypotheses can fully account for our observed results, what are the alternatives? It seems plausible that the different call types have specific meaning. Indeed, several studies have shown a distinction between 50-kHz flat and frequency-modulated calls, based on both behavioral context and pharmacological treatment [3, 29, 31, 32]. Further, vocalizations may be temporally locked to on-going behavior on a sub-second time scale. Specifically, during juvenile play, Himmler et al. [48] showed that 50-kHz calls were more frequent in a 300 ms window before contact is made compared to a 300 ms window after contact is terminated. Our results show that there is far more detail in both the specificity of calls and to their temporal coupling to behavior than has previously been suspected. The finding that rats emit specific calls tied to their ongoing behavior raises the possibility that such calls may be used to coordinate moment-by-moment social interactions (i.e., to warn a playmate of an impending attack, communicate that the contact is friendly, beg for mercy, etc.). An important caveat is that our findings come from single animals awaiting a play partner, which undermines the idea that the observed vocalizations serve a social role. However, the number of vocalizations increased significantly over 7 days of repeated reunions with a play partner in the test chamber, suggesting that vocalizations may be intended for the soon-to-arrive play partner. To pursue the possible communicative role of specific vocalizations, the positive and negative associations between specific call types and behaviors found in the present study in the absence of a partner need to be compared with the associations that are produced when a social partner is present.

How do our results compare with previous studies? Our results are consistent with the high association between movements and 50-kHz vocalizations reported by Laplagne and Costa [13]. They showed a striking increase in what they called “step” calls during high-speed locomotion. Closer inspection of their examples suggests that their “step” includes Wright et al.’s step, split and complex types. This is entirely consistent with our finding of a strong association between these same three calls and jumps. Takahashi et al. [33] also found a strong relationship between movement and high-frequency calls. In their case, calls above 60 kHz, which included trills, upward ramp and part of the step call were strongly associated with locomotion. Our results agree, but also include several other 50-kHz calls associated with movement including trills-with-jumps, complex, composite and multi-step calls. Contrasting with previous reports in gerbils [53] and rats [2] which claim that vocalizations were frequently tied to the landing of the forepaws after a jump, we find that vocalizations are most often associated with the onset of a movement. Our results agree with the more recent observations of Laplagne and Costa [13], who found a dramatic increase in call density peaking 140 ms before movement onset.

An important issue is whether the results obtained from our analysis method are stable across repeated observations. To this end, we replicated our analysis twice using different testing days and different coders. Based on a subset of data coded by both coders, we found that agreement for behavioral categories was quite high (92%) while agreement on vocal categories was much lower (36%). The issue was that several of the most frequent call types (trills, complex, composite and trill-with-jumps) are highly similar, leading to different decisions by the two coders. By temporarily treating these four categories as identical, we found a much higher agreement between coders (98%). Despite the less-than-perfect agreement between coders, the overall pattern of results is strikingly similar between analyses (compare Fig 3A to 3B), helping to establish that our results are not due to sampling or statistical errors. The slight differences between analysis 1 and analysis 2 may be due to coding differences but also suggest that slightly more observation time may be necessary to obtain truly stable correspondence between vocalizations and behaviors. The limitation here is not minutes of observation, of which we had an easily manageable 24–28 minutes per analysis, but the time it takes to manually identify and categorize behaviors and calls (2946 behaviors and 4272 vocalizations for the combined analyses). Our best guess as to the true call-behavior correspondence is provided by the averaged data from our two analyses (Fig 3C).

As mentioned in the Introduction, it is currently unclear how many call categories should be used in the analysis of rat USVs. Several researchers have broken 50-kHz calls into flat and FM calls [3, 29, 31, 32]. Takahashi et al. [33] found evidence for two categories within the 50-kHz range, a group of 60 kHz calls broadly associated with movement and another of 40 kHz calls associated with feeding. Brudzynski [36] has suggested that four categories are probably sufficient to capture the important distinctions between 50-kHz calls (flats, trills, step-trills or “other”) while Wright et al. [3] found evidence for 14 distinct calls within the 50-kHz range. Our study offers a possible answer to this question. A clustering algorithm was used to identify groups of calls with similar behavioral correlates among the call categories originally identified by Wright et al. [3]. It is perhaps worth emphasizing that our categorization is based on the way the calls are used, not their acoustic similarity. As shown in Fig 5, this clustering algorithm identifies three categories of calls: Multi-part calls (including composite, split, and multi-step) were all associated with running and jumping; calls with trills were associated with slower locomotion; and inverted U and up ramp calls had similar, though less well-defined, behavioral correlates. Step-up calls may form a fourth distinct category associated with walking. The similarities in the usage of the calls we’ve identified are based on objective criteria, although admittedly it is not clear what level of similarity should be used as a criterion. An important caveat is that certain distinctions between calls may become relevant in other behavioral circumstances. For example, flat calls are likely to form a separate behavioral category, such as re-establishing social contact with cage mates [54], but we did not even include them in our clustering because they were so infrequent in our paradigm. Another caveat is that the perceptual similarities among certain call types may have artificially increased our estimates of their behavioral similarities. For example, as revealed by our coder reliability data, calls with trills (trill, trill-with-jumps, complex and flat-trill) were easily confused, reducing our ability to discriminate any differences in their usage. Studies of categorical perception in human speech show that acoustic parameters of a sound do not necessarily predict their perceptual similarity [55]. Hence, we cannot assume that calls that we perceive as similar based on a spectrogram are, in fact, similar to the rat. It remains possible that trills, trills-with-jumps, and complex calls have different meanings that will manifest once a more objective method of vocal categorization is implemented. In sum, our study provides a method to identify groupings of calls but further studies using different behavioral contexts and, perhaps, automated recognition of vocalizations, will be necessary to definitively identify the behaviorally relevant call categories.

## Conclusions

We have presented novel methods for identifying the behavioral correlates between behavioral actions and vocalizations during a behavioral test paradigm in rats (Figs 3 and 4). Our findings provide new clues about how and why rats emit 50kKz vocalizations. The lack of a clear one-to-one relationship between one specific action and one specific call casts doubt on the proposition that FM 50-kHz calls are a byproduct of locomotion. On the other hand, it is difficult to explain why calls meant to convey an affective state would be specifically linked to certain behaviors. If the tight coupling between vocalizations and behavior persist during social interactions, they could be used to signal specific actions, allowing animals to anticipate such things as nose-to-nape contact and react accordingly. The methodology outlined in the present paper could be used to more fully explore the social dimension of 50-kHz calls. In addition, once these findings are validated in different social and non-social contexts, the vocalization-behavior associations may also have practical applications. For example, if particular behavior-vocalization profiles are robust, they could be used to more thoroughly evaluate neurobehavioral disruptions produced by psychotropic drugs and models of mania or depression.
